# Comparison of the Efficacy and Safety of S-1-Based and Capecitabine-Based Regimens in Gastrointestinal Cancer: A Meta-Analysis

**DOI:** 10.1371/journal.pone.0084230

**Published:** 2014-01-02

**Authors:** Xunlei Zhang, Chunxiang Cao, Qi Zhang, Yi Chen, Dongying Gu, Yunzhu Shen, Yongling Gong, Jinfei Chen, Cuiju Tang

**Affiliations:** 1 Department of Oncology, Nanjing First Hospital, Nanjing Medical University, Nanjing, China; 2 Department of Oncology, Nanjing First Hospital, Medical School of Southeast University, Nanjing, China; European Institute of Oncology, Italy

## Abstract

**Purpose:**

Oral fluoropyrimidine (S-1, capecitabine) has been considered as an important part of various regimens. We aimed to evaluate the efficacy and safety of S-1-based therapy versus capecitabine -based therapy in gastrointestinal cancers.

**Methods:**

Eligible studies were identified from Pubmed, EMBASE. Additionally, abstracts presented at American Society of Clinical Oncology (ASCO) conferences held between 2000 and 2013 were searched to identify relevant clinical trials. The outcome included overall survival (OS), progression-free survival (PFS), overall response rate (ORR), disease control rate (DCR) and advent events.

**Results:**

A total of 6 studies (4 RCTs and 2 retrospective analysis studies) containing 790 participants were included in this meta-analysis, including 401 patients in the S-1-based group and 389 patients in the capecitabine-based group. Results of our meta-analysis indicated that S-1-based and capecitabine-based regimens showed very similar efficacy in terms of PFS (HR 0.92, 95% CI 0.78–1.09, *P* = 0.360), OS (HR 1.01, 95% CI 0.84–1.21, *P* = 0.949), ORR (HR 1.04, 95% CI 0.87–1.25, *P* = 0.683) and DCR (HR 1.02, 95% CI 0.94–1.10, *P* = 0.639). There was also no significant difference in toxicity between regimens other than mild more hand–foot syndrome in capecitabine-based regimens.

**Conclusion:**

Both the S-1-based and capecitabine-based regimens are equally active and well tolerated, and have the potential of backbone chemotherapy regimen in further studies of gastrointestinal cancers.

## Introduction

Gastrointestinal cancers, especially gastric and colorectal cancers, are a major global health concern. Previous studies suggest that factors such as dietary, lifestyle, other personal exposures, and genetic factors might increase the susceptibility to developing gastrointestinal cancer [1]. Gastric cancer (GC) and colorectal cancer (CRC) are the third and the fourth common cancers in the world behind lung cancer and breast cancer, and are also the major causes of cancer-related deaths globally [2], [3]. The most commonly used regimens for GC are combination chemotherapy consisting of a fluoropyrimidine (5-fluorouracil or oral fluoropyrimidine, 5-Fu) plus a platinum agent with or without docetaxel or anthracyclines [4], [5], [6], [7]. Doublet combination chemotherapy plus targeted agents is a widely used treatment strategy for the first-line treatment of patients with CRC, and oxaliplatin plus either fluorouracil or capecitabine is one of the reference doublet cytotoxic chemotherapy strategies [8], [9].

From the above-mentioned,fluoropyrimidines have remained the most commonly prescribed agents for gastrointestinal cancers in various settings. 5­FU administered as a continuous infusion by a portable pump provides prolonged exposure and modest improvement in efficacy. However, the infusion is inconvenient and unsafe, for it can plague with more catheter­related events hematological toxicity and hand–foot syndrome [10], [11].

For this reason, oral fluoropyrimidine (S-1, capecitabine) has been studied as a substitute for continuous infusion of 5-FU. S-1 is a novel oral fluoropyrimidine consisting of a 5-FU prodrug, tegafur, and the dihydropyrimidine dehydrogenase inhibitor, 5-chloro-2, 4-dihydroxypyridine and the orotate phosphoribosyl transferase inhibitor, potassium oxonate, which suppresses the gastrointestinal toxicity of tegafur [12]. The FLAGS trial revealed a similar efficacy and better toxicity profile of S-1 compared to infusional 5-FU [13]. Capecitabine is an oral fluoropyrimidine, which is metabolized primarily in the liver and converted in tumor tissues to 5-FU by the enzyme thymidine phosphorylase, which is present in higher concentrations in tumor cells than in normal cells. Additionally, meta-analysis of 2 trials showed that OS was superior in the patients treated with capecitabine combinations than in the patients treated with 5-FU combinations [14]. By virtue of their oral formulations, promising efficacy, and favourable toxicity profiles, S-1 and capecitabine may be particularly attractive for elderly cancer patients [15].

Previous study compared the efficacy and safety of S-1 and capecitabine in patients with GC, showing that there were no significant differences in objective response rate (ORR), progression-free survival (PFS) and overall survival (OS) between the S-1 and capecitabine groups, although some results showed capecitabine has a slightly longer OS (statistically not significant) in addition to a higher rate of adverse events such as the hand–foot syndrome and diarrhea[15], [16], [17], [18]. however, when compared in CRC, Hong et al. found S-1 group have a nearly 2 months longer in PFS than capecitabine group from a phase III trial, while Zang et al. reported capecitabine group have a 3 months longer in OS from a newest phase II trial [19].

In gastrointestinal cancer, several randomized controlled trials (RCTs) and retrospective research, comparing S-1 with capecitabine in mono or combined therapy, have been conducted, with not consistent completely, none of which have allowed the definite conclusions about the efficacy and safety of these two therapies. Additionly, there has been no meta-analysis to detect the treatment differences with greater power of statistical comparisons. Therefore, we conducted a meta-analysis to give an overview of the results of all eligible studies with the aim of investigating the differences of the efficacy and safety between S-1 and capecitabine groups in gastrointestinal cancers.

## Methods

### Search strategy

We did a comprehensive search of citations from Pubmed, EMBASE from April 1966 to July 2013 using the following terms, which included in their titles, abstracts, or keyword lists: ‘S-1’, ‘capecitabine’, ‘gastric cancer’, ‘colorectal cancer’, ‘gastrointestinal cancer’ without any language restriction. In addition, all abstracts and virtual meeting presentations from the American Society of Clinical Oncology (ASCO) conferences held between 2000 and 2013 were also searched for relevant research. We included studies that reported the patient numbers and characteristics, treatment regimen and study outcome including efficacy and safety. We resolved disagreements by consensus or by a third reviewer if necessary.

### Study selection

Studies that met the following criteria were included in the meta-analysis: (i) patients with gastrointestinal cancer at baseline; (ii) studies comparing S-1-based therapy with capecitabine -based therapy: mono or combined chemotherapy with S-1 versus capecitabine and not confounded by additional agents or interventions (i.e. in the combination chemotherapy, the control and experimental arms had to differ only by S-1 and capecitabine components); (iii) randomised controlled trials (RCTs), quasi-RCTs, and retrospective or prospective controlled studies. Two reviewers independently assessed each study for inclusion using a standardized form with eligibility criteria. Each study was fully examined to eliminate duplicates.

### Data extraction

Two reviewers extracted data from each report independently and reached a consensus on all items. The following data were retrieved: study authors, publication year, phase design, number of patients, sex, median age, cancer type, chemotherapy regimen, median OS, PFS, and adverse events (AEs). Hazard ratios (HRs) for OS and PFS were extracted directly from the original studies or were estimated indirectly by reading off survival curves as suggested by Parmar and colleagues [20].

### Statistical analysis

OS and PFS rate was used as the primary outcome measure. Secondary outcome measures evaluated were ORR (number of partial and complete responses), disease control rate (DCR: number of partial and complete responses and stable disease) and toxicities (published by the authors with the most frequently reported events analyzed) [21], [22]. Statistical analysis of the overall hazard ratio (HR) and the 95% CIs for OS and PFS, the risk ratio (RR) for ORR, DCR and AEs was calculated using STATA version 10.0 (Stata Corporation, College Station, Texas, USA). We also compared the pooled estimates of the above efficacy outcomes for subpopulations stratified by age, combined medicine, treatment schedule, trial type and cancer type. An HR<1 indicates a favorable outcome in the S-1-based regimens for OS and PFS. An RR>1 favors S-1-based group for response rate, or indicates more toxicity or treatment-related deaths in the S-1-based group. The efficacy and safety of pooled estimates were calculated using the fixed-effects model first [22]. If any heterogeneity existed, a random-effects model was applied in a sensitivity analysis. The traditional Q test and the I^2^ statistic were used to evaluate heterogeneity and a P<0.1 was considered as heterogeneity between studies. The presence of publication bias was evaluated by using the Begg's and Egger's tests [23], [24]. A 2-tailed P value of less than 0.05 was judged as statistically significant.

## Results

### Search results and description of the studies

**Figure 1:**
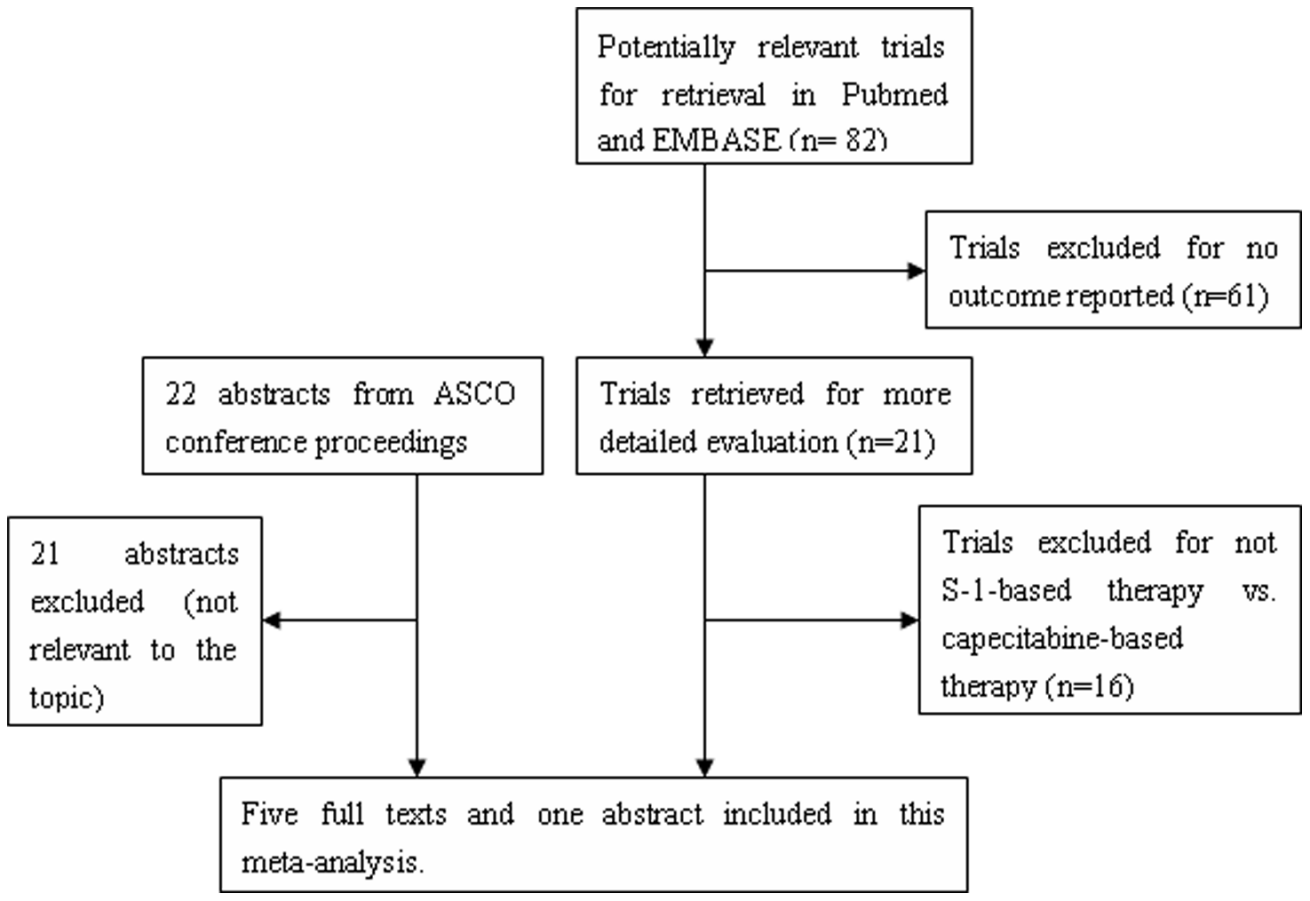
Flow diagram summarizing the search strategy.

The study flow diagram is shown in Figure 1. In total, 6 studies [15], [16], [17], [18], [19], [25] fulfilled the inclusion criteria of this meta-analysis, with four studies on GC and two studies on CRC. Among the selected studies, 4 were prospective clinical trials (3 randomized controlled phase II trial, 1 randomized controlled phase III trial) and 2 were retrospective analysis studies. All the patients included in our pooled analysis were Asian population.

A total of 790 participants were included in this meta-analysis, including 401 patients in the S-1-based group and 389 patients in the capecitabine-based group. Patient enrollment ranged between 72 and 340, and median age of patients ranged from 60 to 74. The used drugs were S-1, capecitabine, cisplatin, and oxaliplatin, and regimens were similar with respect to doses in every trial. The baseline characteristics of the 6 studies were summarized in Table 1. All the studies included in the meta-analysis were reasonably well conducted and had balanced populations.

**Table 1:** Characteristics of literatures included in the meta-analysis.

Author	Year	Country	Study design	Cancer type	Chemotherapy regimen	Age S/C (Year)	Number S/C	Median PFS S/C (Months)	Median OS S/C (Months)
Kim[16]	2012	korea	RCT II	GC	S−1+Oxaliplatin vs. capecitabine+ Oxaliplatin 2 W/cycle	60/61	65/64	6.2/7.2	12.4/13.3
Lee[15]	2008	korea	RCT II	GC	S−1 vs. Capecitabine 4 W/cycle	71/71	45/46	4.2/4.7	8.1/9.5
Seol[17]	2009	korea	Retrospective	GC	S−1+Cisplatin vs. capecitabine+Cisplatin 2 W/cycle	73/74	32/40	5.4/5.9	9.6/10.8
Shitara[18]	2012	Japan	Retrospective	GC	S−1+cisplatin vs. Capecitabine+cisplatin 4 W/cycle	61/65	50/26	5.8/5.2	13.8/13.5
Zang[25]	2012	korea	RCT II (Abstract)	CRC	S−1+Oxaliplatin vs. capecitabine +Oxaliplatin 2 W/cycle	67	41/41	6.6/8.1	19/22
Hong[19]	2012	korea	RCT III	CRC	S−1+Oxaliplatin vs. capecitabine+ Oxaliplatin 2 W/cycle	61/60	168/172	8.5/6.7	21.2/20.5

GC, gastric cancer; CRC, colorectal cancer; OS, overall survival; PFS, progression-free survival; RCT: randomized controlled trial; S, S−1; C, capecitabine.

### Efficacy comparison

#### Progression-free survival (PFS) and overall survival (OS)

**Figure 2:**
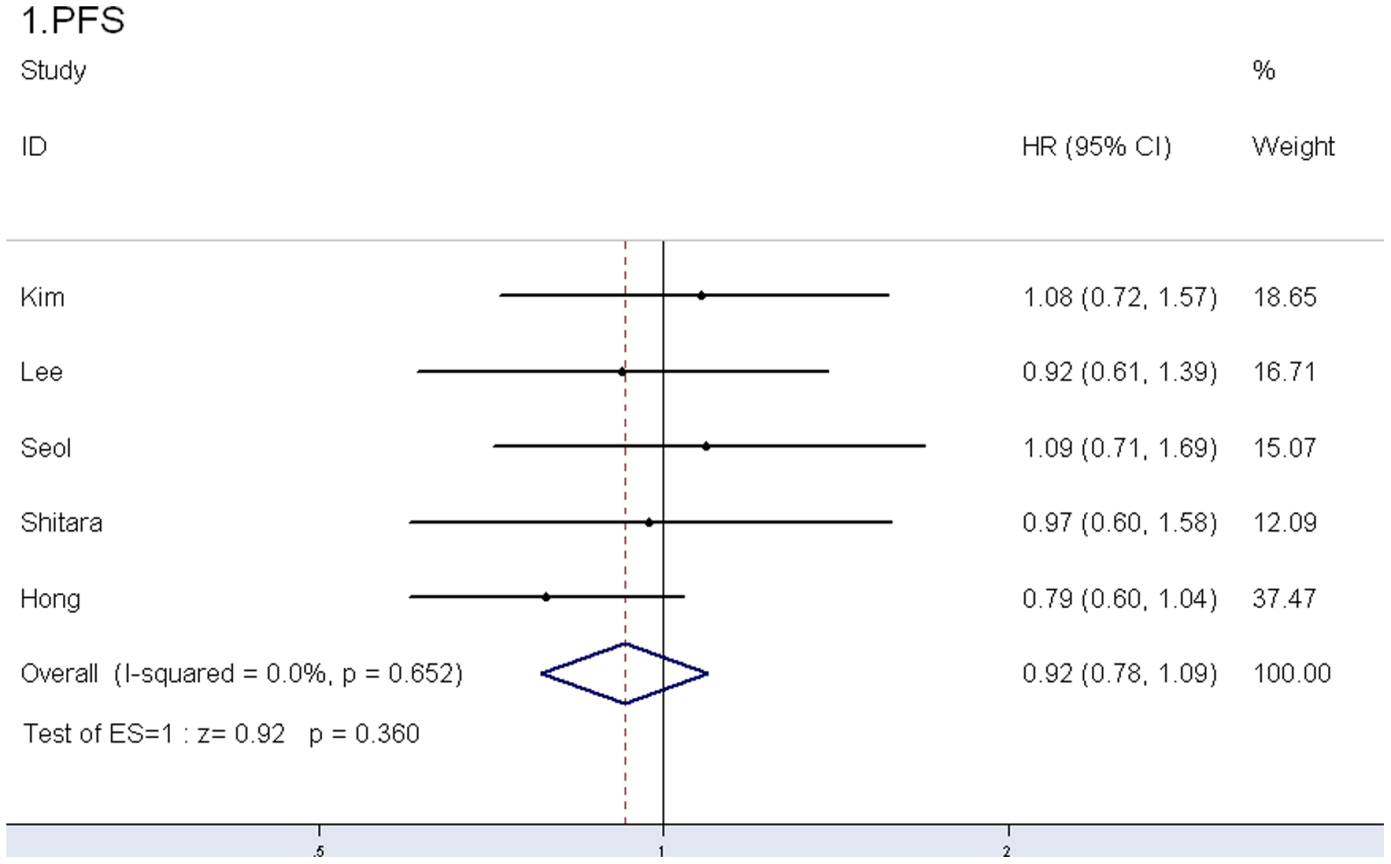
Fixed-effects model of hazard ratio (95% confidence interval) of PFS associated with S-1-based therapy compared with capecitabine-based therapy.

**Figure 3:**
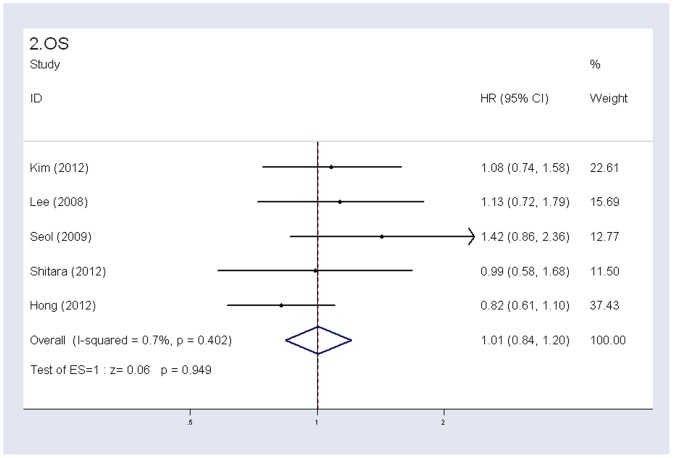
Fixed-effects model of hazard ratio (95% confidence interval) of OS associated with S-1-based therapy compared with capecitabine-based therapy.

5 of the 6 studies [15], [16], [17], [18], [19] with sufficient PFS and OS data were included in the meta-analysis (Figure 2, Figure 3). Our results showed that there was no significant difference in PFS or OS between S-1-based group and capecitabine-based group (PFS: HR 0.92, 95% CI 0.78–1.09, *P* = 0.360, I^2^ = 0%; OS: HR 1.01, 95% CI 0.84–1.21, *P* = 0.949, I^2^ = 0.7%) (Table 2).

**Table 2:** Hazard ratios, *P* value, and heterogeneity for PFS and OS in the stratified analyses.

Efficacy	n	PFS					OS				
		HR	*P*	*P*_H_	*I* ^2^/%	HW	HR	*P*	*P*_H_	*I* ^2^/%	HW
**All**	5	0.92(0.78,1.09)	0.360	0.652	0.0	100	1.01(0.84,1.21)	0.949	0.402	0.7	100
**type**											
GC	4	1.02(0.82,1.26)	0.886	0.929	0.0	62.53	1.14(0.91,1.43)	0.271	0.783	0.0	62.57
CRC	1	0.79(0.60,1.04)	0.093	N/A	N/A	37.47	0.82(0.61,1.10)	0.187	N/A	N/A	37.43
**age**											
<70	3	0.89(0.73,1.09)	0.274	0.409	0.0	68.21	0.92(0.75,1.14)	0.456	0.511	0.0	71.54
≥70	2	1.00(0.74,1.34)	0.984	0.578	0.0	31.79	1.25(0.89,1.76)	0.193	0.510	0.0	28.46
**combine**											
Oxa	2	0.88(0.70,1.10)	0.250	0.199	39.3	67.38	0.91(0.72,1.15)	0.425	0.261	20.8	71.21
Cis	2	0.93(0.77,1.11)	0.835	0.725	0.0	32.62	1.20(0.83,1.73)	0.336	0.335	0.0	28.79
**study**											
RCT	3	0.89(0.73,1.08)	0.230	0.429	0.0	72.83	0.95(0.77,1.17)	0.638	0.376	0.0	75.73
RS	2	1.04(0.75,1.43)	0.835	0.725	0.0	27.17	1.01(0.84,1.21)	0.336	0.335	0.0	24.27
**schedule**											
2 week	3	0.92(0.75,1.12)	0.400	0.299	17.2	71.20	0.98(0.80,1.22)	0.878	0.155	46.4	72.81
4 week	2	0.94(0.69,1.29)	0.702	0.870	0.0	28.80	1.07(0.76,1.51)	0.707	0.711	0.0	27.19

HR, hazard ratio; *P*_H_, heterogeneity *P*; GC, gastric cancer; CRC, colorectal cancer; OS, overall survival; PFS, progression-free survival; RCT, randomized controlled trial; RS, Retrospective study;

In the subgroup analysis by cancer types, no significant difference was observed in PFS or OS between S-1-based and capecitabine-based regimens in GC group (PFS: HR 1.02, 95% CI 0.82–1.26, *P* = 0.886, I^2^ = 0%; OS: HR 1.14, 95% CI 0.91–1.43, *P* = 0.271, I^2^ = 0%). Similar results were observed in CRC group. Additionally, in the stratified analysis by age, combined medicine, treatment schedule and trial type, the results of predefined clinical subgroup analyses for PFS and OS were generally consistent with the results found in all patients (statistically not significant). Besides, there was no heterogeneity observed (Table 2).

#### Objective response rate (ORR) and disease control rate (DCR)

**Figure 4:**
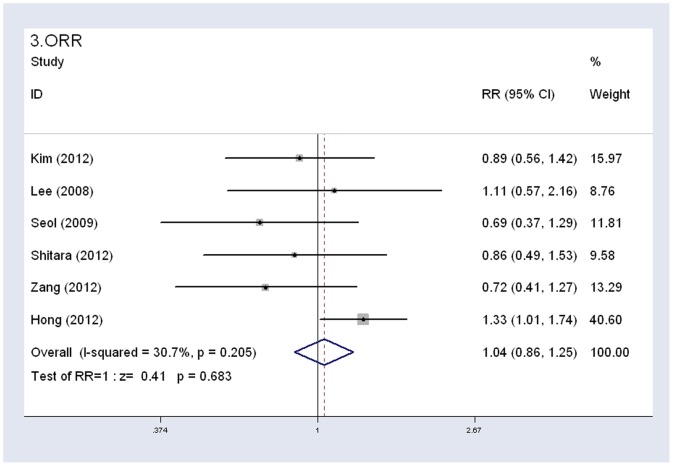
Fixed-effects model of hazard ratio (95% confidence interval) of ORR associated with S-1-based therapy compared with capecitabine-based therapy.

**Figure 5:**
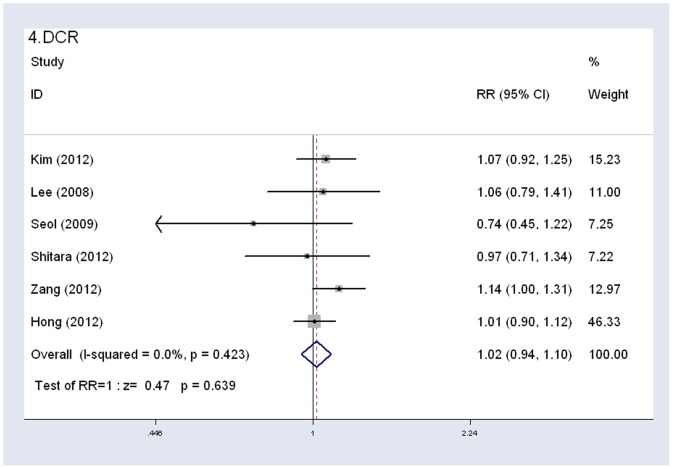
Fixed-effects model of hazard ratio (95% confidence interval) of DCR associated with S-1-based therapy compared with capecitabine-based therapy.

All six studies reported ORR and DCR data. The ORR was 40.2% (146 of 363 patients) in the S-1-based group and 38.3% (133 of 347 patients) in the capecitabine-based group. The DCR was 78.5% (285 of 363 patients) in the S-1-based group and 76.4% (265 of 347 patients) in the capecitabine-based group. Though the comparison of S-1 with capecitabine showed that S-1-based group had a slightly higher ORR and DCR, the pooled RR for overall response rate and disease control rate showed no statistically significant difference between the two groups (ORR: HR 1.04, 95% CI 0.87–1.25, *P* = 0.683, I^2^ = 30.7%; DCR: HR 1.02, 95% CI 0.94–1.10, *P* = 0.639, I^2^ = 0%) (Figure 4, Figure 5). In the subgroup analysis by combined medicine, no significant difference was observed in ORR or DCR between S-1 combined oxaliplatin and capecitabine combined oxaliplatin regimens. Similar results were observed between S-1 combined cisplatin and capecitabine combined cisplatin regimens, which indicated that S-1 was comparable to capecitabine in the two most commonly used regimens in gastrointestinal cancers (Table 3).

**Table 3:** Hazard ratios, *P* value, and heterogeneity for ORR and DCR in the stratified analyses.

Efficacy	n	ORR					DCR				
		RR	*P*	*P*_H_	*I* ^2^/%	HW	RR	*P*	*P*_H_	*I* ^2^/%	HW
**All**	6	1.04(0.87,1.25)	0.683	0.205	30.7	100	1.02(0.94,1.10)	0.639	0.423	0.0	100
**type**											
GC	4	0.88(0.66,1.16)	0.363	0.796	0.0	46.12	0.99(0.86,1.14)	0.898	0.469	0.0	40.70
CRC	2	1.18(0.93,1.50)	0.185	0.057	72.3	53.88	1.04(0.95,1.13)	0.416	0.136	55.1	59.30
**age**											
<70	4	1.08(0.89,1.32)	0.438	0.145	44.5	79.43	1.04(0.96,1.12)	0.330	0.468	0.0	81.75
≥70	2	0.87(0.55,1.37)	0.546	0.314	1.2	20.57	0.93(0.72,1.20)	0.581	0.213	35.6	18.25
**combine**											
Oxa	3	1.11(0.90,1.38)	0.330	0.094	57.8	76.55	1.05(0.97,1.13)	0.267	0.330	9.9	83.75
Cis	2	0.77(0.51,1.18)	0.227	0.607	0.0	23.45	0.86(0.64,1.14)	0.292	0.331	0.0	16.25
**study**											
RCT	4	1.11(0.91,1.37)	0.309	0.192	36.7	78.61	1.05(0.97,1.13)	0.250	0.533	0.0	85.53
RS	2	0.77(0.51,1.18)	0.227	0.607	0.0	21.39	0.86(0.64,1.14)	0.292	0.331	0.0	14.47
**schedule**											
2 week	4	1.05(0.86,1.29)	0.622	0.080	55.6	81.66	1.02(0.94,1.10)	0.673	0.183	38.2	81.79
4 week	2	0.98(0.63,1.52)	0.931	0.577	0.0	18.34	1.02(0.83,1.27)	0.834	0.706	0.0	18.21

HR, hazard ratio; *P*_H_, heterogeneity *P*; GC, gastric cancer; CRC, colorectal cancer; ORR, objective response rate; DCR, disease control rate; RCT, randomized controlled trial; RS, Retrospective study.

Additionally, in the stratified analysis by age, cancer type, treatment schedule and trial type, the results of ORR and DCR were generally consistent with the results found in all patients (statistically not significant). Besides, there was no heterogeneity observed (Table 3).

### Safety

Safety-related information was reported in all the 6 studies. The common AEs were anaemia, neutropenia, thrombocytopenia, asthenia, anorexia, nausea and neuropathy, which were experienced by nearly half of the patients both in S-1-based and capecitabine-based group. Anorexia was the most common AE both in the two groups (67% in S-1-based group and 59% in capecitabine-based group) and happened slightly more frequently in S-1-based regimens (RR 1.13, 95% CI 1.01–1.27, *P* = 0.034). As anticipated, the frequency of hand foot syndrome (HFS) was 10% in S-1-based group and 33% in capecitabine-based group, with a significant difference between them (RR 0.30, 95% CI 0.22–0.42, *P*<0.001).

As anticipated, the frequency of hand foot syndrome (HFS) in capecitabine-based group was significantly more common than in S-1-based group (10% in S-1-based, 33% in capecitabine-based, RR 0.30, 95% CI 0.22–0.42, *P*<0.001). Similar results were observed in the two groups when comparing the Grade 3 or 4 HFS (0.3% in S-1-based, 3% in capecitabine-based, RR 0.23, 95% CI 0.07–0.78, *P* = 0.019). No significant differences regarding the occurrence of other AEs at any grade was found between the two groups (Table 4).

**Table 4:** Summary of adverse events.

All grade AEs	N	S-1 N/T	%	Capecitabine N/T	%	RR(95%CI)	*P* value	Grade 3–4 AEs	N	S-1 N/T	%	Capecitabine N/T	%	RR(95%CI)	*P* value
Anaemia	6	206/399	51.6	178/381	46.7	1.10(0.99,1.22)	0.090	Anaemia	6	35/399	8.8	23/381	6.0	1.39(0.85,2.28)	0.195
Neutropenia	6	183/399	45.9	172/381	45.1	0.98(0.84,1.14)	0.767	Neutropenia	6	76/399	19.0	54/381	14.2	0.92(0.45,1.86)	0.806
Leukopenia	4	75/326	23.0	65/300	21.7	0.95(0.75,1.21)	0.697	Leukopenia	4	10/326	3.1	5/300	1.7	1.70(0.59,4.91)	0.328
Thrombocytopenia	6	183/399	45.9	151/381	39.6	1.09(0.82,1.45)	0.570	Thrombocytopenia	6	56/399	14.0	33/381	8.7	1.32(0.55,3.14)	0.533
Asthenia	5	183/358	51.1	167/340	49.1	1.07(0.93,1.24)	0.365	Asthenia	5	19/358	5.3	20/340	5.9	0.92(0.50,1.69)	0.788
Anorexia	5	239/358	66.8	202/340	59.4	1.13(1.01,1.27)	0.034	Anorexia	5	31/358	8.7	16/340	4.7	1.67(0.94,2.98)	0.081
Nausea	5	193/358	53.9	169/340	49.7	1.08(0.93,1.24)	0.322	Nausea	5	19/358	5.3	14/340	4.1	1.17(0.60,2.28)	0.643
Vomiting	5	116/358	32.4	108/364	29.7	1.09(0.88,1.35)	0.440	Vomiting	5	8/358	2.2	11/364	3.0	0.77(0.32,1.83)	0.554
Diarrhoea	5	128/358	35.8	109/340	32.1	1.13(0.91,1.39)	0.265	Diarrhoea	5	25/356	7.0	16/340	4.7	1.49(0.81,2.74)	0.206
Stomatitis	4	88/293	30.0	73/276	26.4	0.91(0.40,2.08)	0.829	Stomatitis	4	3/293	1.0	1/276	0.4	1.51(0.29,7.78)	0.622
Neuropathy	4	182/307	59.3	184/311	59.2	0.98(0.87,1.10)	0.709	Neuropathy	4	19/307	6.2	14/311	4.5	1.34(0.69,2.61)	0.395
Hand foot syndrome	6	40/399	10.0	127/381	33.3	0.30(0.22,0.42)	<0.001	Hand foot syndrome	6	1/399	0.3	12/381	3.1	0.23(0.07,0.78)	0.019

AEs, adverse events; CI, confidence interval; N/T, the number of adverse reactions/the total number of patients; RR, risk ratio.

### Publication bias

**Figure 6:**
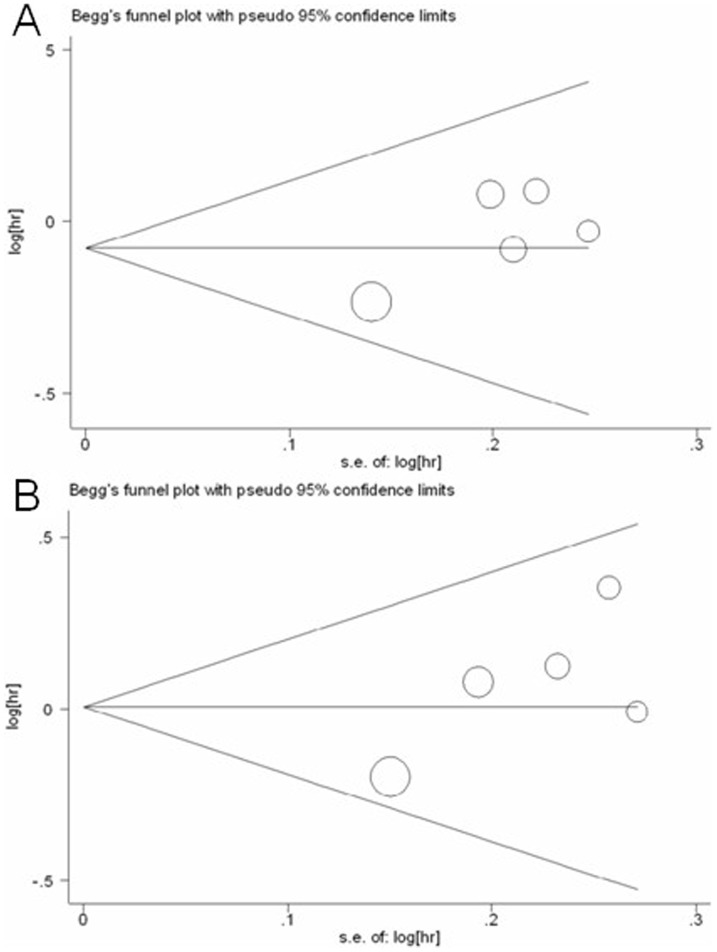
Begg's funnel plot of publication bias test. (A) PFS; (B) OS. Each point represents a separate study for the indicated association. Log (OR), natural logarithm of OR. Horizontal line, mean effect size.

Begg's funnel plot and Egger's test were performed to assess the publication bias of literatures. The shapes of the funnel plots did not reveal any evidence of obvious asymmetry (*P* = 0.806 for PFS, *P* = 0.462 for OS, Figure 6). Then, Egger's test was used to provide statistical evidence of funnel plot symmetry. The results still did not suggest any evidence of publication bias (*P* = 0.098 for PFS, *P* = 0.122 for OS, respectively).

## Discussion

This is the first meta-analysis to estimate the relative efficacy and safety of two new oral fluoropyrimidines, S-1 and capecitabine. Our results indicated that S-1-based and capecitabine-based regimens showed very similar efficacy in terms of PFS, OS, ORR and DCR. There was also no significant difference in toxicity between regimens other than mild more hand–foot syndrome in capecitabine-based regimens. In conclusion, both the S-1-based and capecitabine-based regimens are equally active and well tolerated, and have the potential of backbone chemotherapy regimen in further studies of gastrointestinal cancers.

After years of argument about the utility of chemotherapy for gastrointestinal cancer, extensive clinical research contributed to the optimization of fluoropyrimidines administration, with oral S-1 and capecitabine emerging as the standard therapy in advanced gastrointestinal cancer. Since S-1 and capecitabine offered the advantages of simplicity and convenience over the traditional 5-FU, they have opened new perspectives for improving survival of patients with gastrointestinal cancer.

The findings of the Japan Clinical Oncology Group (JCOG) 9912 trial that compared fluorouracil alone versus irinotecan plus cisplatin versus S-1 alone, suggested that S-1 was no worse than fluorouracil or irinotecan plus cisplatin in advanced gastric cancer (AGC)[26]. Additionally, S-1 combined with cisplatin (SP), showed superior efficacy to S-1 alone in the SPIRITS trial [6] and has now became the standard chemotherapy for AGC in Japan. However, in a large, non-Japanese, phase III trial (the First-Line Advanced Gastric Cancer Study; FLAGS trial), SP did not show superiority compared with 5-FU plus cisplatin, although exploratory analysis demonstrated significant non-inferiority with fewer toxic effects [13]. Kang et al. evaluated capecitabine plus cisplatin (XP) versus 5-FU plus cisplatin, showing significant non-inferiority in the median PFS showed [7]. In the REAL-2 study, statistical non-inferiority for OS was achieved for comparisons of capecitabine versus 5-FU [27]. Additionally, meta-analysis of these two trials showed that OS was superior in the capecitabine-based regimens than 5-FU-based regimens [14]. On the basis of these results, XP regimen is now considered one of the standard chemotherapy for AGC, and recently two global studies of molecular targeting agents each adopted XP regimen as the reference arm [28], [29].

Recently, several studies had focused on the difference between S-1-based and capecitabine-based regimens. A previous phase II study of capecitabine monotherapy in Japan showed an ORR of 23% [30], which seemed to be lower than that of S-1 [31]. However, Lee et al. [15] performed a randomized phase II study of monotherapy of S-1 and capecitabine for elderly AGC patients, and reported similar efficacies and safety for them. In the study by Hong in colorectal cancer, S-1 combined with oxaliplatin (SOX) was non-inferior to standard capecitabine combined with oxaliplatin (CapeOX) in terms of PFS, which is still regarded as one of the reference doublet cytotoxic chemotherapy in many countries, and showed improvements in ORR, incidences of grade 3–4 neutropenia, thrombocytopenia, and diarrhoea were higher in the SOX group than in the CapeOX group[19]. The limited number of trials, with dissimilar criteria, methodologies, and evaluation standards used, had likely resulted in inconsistent outcomes. To comprehensively assess the advantages and disadvantages of S-1-based and capecitabine-based therapy for patients with gastrointestinal cancer, we undertook a meta-analysis of published data from all the correlated studies.

Our results showed that there was no significant difference in terms of PFS, OS, ORR or DCR between S-1-based regimens and capecitabine-based regimens, and sharing very similar efficacy. In the subgroup analysis by cancer types, no significant difference was observed in PFS or OS between the two groups which were consistent with the included studies. When comparing the efficacy differed by age, Lee et al. have reported similar efficacies and safety for elderly AGC patients between the two regimens [15]. Similar results were observed not only in the elderly group, but also in the younger group. Oral fluoropyrimidines combined with cisplatin or oxaliplatin were most common regimes in the gastrointestinal cancers. Recently research focused on these regimens demonstrated SOX and CAPOX, SP and XP were equally active and well tolerated in advanced gastrointestinal cancers [16], [17], [18], [19]. In the subgroup analysis by combined medicine in our meta-analysis, S-1 showed the similar efficacy with capecitabine when combined with cisplatin or oxaliplatin in GC and CRC.

With regard to safety profile, our analysis suggested that the profile of toxicity associated with both S-1-based therapy and capecitabine-based therapy was equivalent, although a higher incidence of hand–foot syndrome was documented in the capecitabine -based group. Grade 1 or 2 hand–foot syndrome was generally manageable with topical ointments or adequate dose reduction [7]. The rate of grade 3 or 4 hand–foot syndrome in capecitabine -based group was 3% in our pooled analysis, which was lower than reported in a previous study(11–17% in Westerners)[32] suggesting ethnic differences existed. In contrast, toxic effects of S-1 have been reported to be more severe in patients from the USA than in Asian patients [33], [34], [35]. Besides, more S-1-treatment -related deaths have also been mentioned to occur in patients from the USA than Asia [36], resulting in different recommended doses in these populations. These findings warrant careful evaluation of patients appropriate for the regimen.

These two types of fluoropyrimidine have some different characteristics in the mechanism of their antitumor effect. Results from subset analysis of the FLAGS trial and JCOG9912 showed that S-1 was better than 5-FU in patients with gastric cancer associated with high dihydropyrimidine dehydrogenase (DPD), which was found more commonly in diffuse-type tumors than in intestinal-type tumors[37]. Expression of TP is reported to be lacking of association with the efficacy of S-1 or 5-FU in gastric cancer [38] and colorectal cancer [39], [40]. High TP expression in CRC is reported to be associated with higher efficacy of capecitabine-based therapy [41]. Therefore, the biomarkers DPD and TP may be candidates to select whether S-1 or capecitabine be suitable for each patient.

It is important to note the limitations of the present study. First, as with any meta-analysis, the results were affected by the quality of the individual studies. Four of the studies in our meta-analysis were RCTs and two were retrospective studies, while one abstract from ASCO conferences. Insufficient amount of data from abstract might potentially limit detection of the difference, and populations from retrospective studies might contain uncontrolled and potentially heterogeneous. Second, this meta-analysis was not based on individual patient data, which might overestimate treatments effects and preclude a more comprehensive analysis such as adjusting for baseline factors (ECOG status) and other differences that existed between the trials from which the data were pooled. Third, these studies were conducted at major academic institutions among patients with adequate major organ function and might not reflect the general patient population in the community or patients with organ dysfunction. Finally, all of the studies included in this analysis were from Asia, the results need confirmation in the West for the differences of efficacy and safety differed by ethnicities.

In conclusion, this is the first meta-analysis focused on the comparison of the efficacy and safety of S-1-based and capecitabine-based regimens. Stratified analyses were conducted, and the results were consistent with the previous studies. Additionally, no publication biases were detected, which indicated that the results may be unbiased. Our meta-analysis suggests that both the S-1-based and capecitabine-based regimens are equally active and well tolerated, and have the potential of backbone chemotherapy regimen in further studies of gastrointestinal cancers. More high-quality RCTs and Western studies are needed to confirm these findings. Further investigations are also needed to clarify the potential predictive factors for drug selection and to establish the effectiveness of various combinations, including molecular targeted agents.

## Supporting Information

Checklist S1 A PRISMA checklist for this meta-analysis.
(DOC)
